# Psychological Aspects of Bariatric Surgery as a Treatment for Obesity

**DOI:** 10.1007/s13679-017-0242-2

**Published:** 2017-02-27

**Authors:** Sandra Jumbe, Claire Hamlet, Jane Meyrick

**Affiliations:** 1 Centre for Primary Care and Public Health, Blizard Institute, Queen Mary University of London, Yvonne Carter Building, 58 Turner Street, London, E1 2AB UK; 2 Centre for Appearance Research, Department of Health and Social Sciences, University of the West of England, Frenchay Campus, Coldharbour Lane, Bristol, BS16 1QY UK; 3 Department of Health and Social Sciences, University of the West of England, Frenchay Campus, Coldharbour Lane, Bristol, BS16 1QY UK

**Keywords:** Bariatric surgery, Psychological health, Postoperative outcomes, Obesity

## Abstract

**Purpose of Review:**

Little is known about the psychological effects on life after bariatric surgery despite the high prevalence of psychological disorders in candidates seeking this procedure. Our review discusses the literature around the psychological impact of bariatric surgery, exploring whether the procedure addresses underlying psychological conditions that can lead to morbid obesity and the effect on eating behaviour postoperatively.

**Recent Findings:**

Findings show that despite undisputed significant weight loss and improvements in comorbidities, current literature suggests some persisting disorder in psychological outcomes like depression and body image for patients at longer term follow-up, compared to control groups. Lack of postoperative psychological monitoring and theoretical mapping limits our understanding of reasons behind these findings.

**Summary:**

Reframing bariatric approaches to morbid obesity to incorporate psychological experience postoperatively would facilitate understanding of psychological aspects of bariatric surgery and how this surgical treatment maps onto the disease trajectory of obesity.

## Introduction

Obesity is a major health problem worldwide and has reached epidemic proportions in both developed and developing countries [1, 2] making it an extremely important public health issue. Clinically defined in terms of body mass index (BMI), a person is considered obese if their BMI is above 30 kg/m^2^ [3, 4]. Evidence shows obesity as a major risk factor for significant morbidity and mortality [5] including diabetes mellitus, cardiovascular disease [6], non-alcoholic fatty liver disease [7], reduced lung function [8–10] and increased risk of cancers [11, 12]. The condition can also present negative psychological impact resulting in social stigma, mental health and self-esteem issues [13, 14], and poorer quality of life [15].

Bariatric surgery defines a group of surgical procedures performed to facilitate weight loss; open or laparoscopic roux-en-Y gastric bypass (RYGB), sleeve gastrectomy (SG) and adjustable gastric banding (AGB) being the most commonly performed procedures worldwide [16, 17]. There has been an increasing amount of evidence for bariatric surgery as a more effective treatment for morbid obesity [2, 18, 19] compared to dietary advice, exercise, lifestyle changes and medication. In particular, the procedure is more effective in achieving significant weight loss, longer term maintenance, improvements in co-morbidities and reductions in mortality such as physical activity and diet [20, 21]. This growing evidence of the benefits of bariatric surgery has contributed to increased popularity of the procedure over the last decade [22] and led some obesity experts to see the procedure as the solution to the looming obesity epidemic [23]. However, there has been a limited focus in research around the psychological outcomes of bariatric surgery [24]. It is important to consider the psychological effects of these procedures not only in order to optimise their outcome but also to frame the condition in terms of its psychological context, to better understand what leads to morbid obesity and therefore what can prevent or treat the condition.

## Theoretical Models in the Aetiology of Obesity

Currently, there is a general lack of theoretical understanding of what causes and maintains morbid obesity [25] as the aetiological basis of the behaviours that lead to obesity are very complex, including a combination of psychosocial, environmental and biological influences [26, 27]. The question for health psychologists is whether physiological approaches to weight loss, such as bariatric surgery, need to also address underlying psychological conditions that can lead to morbid obesity. Crucially, psychological issues can lead to individuals experiencing difficulty controlling their food consumption and exercising adequately [28, 29•], resulting in the onset and maintenance of obesity, but what is the mechanism behind this pathway?

Theoretical models of the condition are not well developed, but a few useful approaches are covered here. In the realm of addiction theory, obesity has been conceptualised as a consequence of addictive behaviour akin to substance abuse. For instance, an individual is seen as having an ‘impaired control over a reward-seeking behaviour (usually drug-taking) from which harm ensues’ which presents itself in varying extents [30, 31]. Addictive drugs are said to hijack the brain’s reward system by binding to receptor sites that produce intense feelings of pleasure [32]. When this approach is transposed in the context of obesity, highly palatable (or junk) foods are framed as the ‘drugs’ that takeover the individual’s brain reward system, leading to weight gain due to continuous consumption of the junk food, reinforced by pleasurable sensations following dopamine release over time [33].

However, addiction has longstanding negative connotations mostly linked to historically social perceptions of drug addicts as deviants to stability and morality [34]. Therefore, the ‘food addict’ label may stigmatise the obese population as disordered individuals, lacking in self-control [35]. Critics of the addiction approach to obesity have importantly questioned the validity of framing food as an illicit drug [36]. Is it useful to label something as socially embedded in this light, particularly when consumption of junk food is not limited to the obese? However, as pointed out by Schulte and colleagues [37•] if certain foods (e.g. highly processed) are addictive, the identification of possible risk factors for food addiction is key to recognising this. Importantly, this theoretical approach frames behaviour as changeable because internal and external environments directly influence it in a continuously erratic manner [31]. This aptly reflects the variable nature of eating behaviour. Moreover, it shows how challenging it is to tackle overeating and inactivity by using approaches like gradual decision making and action plans, which assume that behaviour change occurs in a linear manner when we know that the factors that trigger motivation to engage in healthy behaviour do not follow a linear fashion [38].

Another theoretical focus in obesity has been on eating as a habitual coping mechanism, drawing attention to the various ways in which past experiences influence our behaviour. Studies indicate that habit strength adds considerably to the extent of variance in healthy eating behaviours across a range of age groups [39]. This implies that a key reason why long-term behaviour change may be difficult for the obese population is that the behaviours that individuals want to change like poor dietary habits and limited physical activity are relatively habitual. Consequently, researchers are working to further understand the role of these concepts in eating behaviours [40, 41] in order to develop effective cue-exposure treatments that potentially decrease food cue reactivity and urges to overeat [42].

In the context of bariatric treatment, research shows that bariatric surgery candidates take longer to be satiated due to slower salivary habituation to food and taste stimuli compared to normal-weight individuals [43] which may influence greater caloric or energy intake. However, bariatric surgery seems to trigger a change in eating behaviour, particularly changes in taste response where patients find sweet and fatty meals less pleasant, which facilitates adoption of healthier foods and subsequent weight loss [44, 45]. Habituation theory therefore may be a useful tool for helping our understanding of eating regulation in morbid obesity and the mechanisms behind postsurgical changes in taste. This could potentially aid development of novel weight loss maintenance interventions following bariatric surgery.

**Fig. 1:**
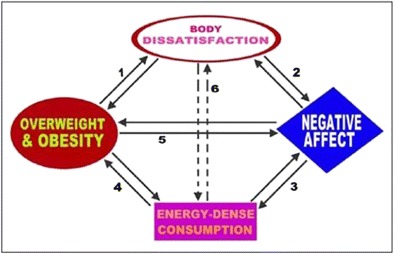
The Circle of Discontent. Reproduced with permission from Marks [29•]

A more recent theoretical model of obesity incorporating biological, psychosocial and environmental factors has been proposed by Marks [29•]. This suggests that the over-consumption of high-calorific, low-nutrient and low-satiating foods, combined with a stressful environment, is the origin of weight gain. Once that weight gain occurs, an individual experiences body dissatisfaction and negative affect leading to continued over-consumption over a prolonged period. This dysfunctional state leaves individuals unable to control weight gain and subsequently forms a vicious ‘Circle of Discontent’ (Fig. 1).

This Circle of Discontent (COD) highlights the important role of body image along its pathway, particularly how general negative affect (depression, low self-esteem) is associated with body dissatisfaction, patterns of consumption and directly with weight status. Although comfort eating may result in temporary reduction in distressed mood, the weight gain that follows may cause a dysphoric mood due to an inability to control one’s distress and subsequent feelings of guilt may reactivate the cycle, leading to a continuous pattern of using food to cope with emotions. This pattern is particularly applicable if there is a genetic predisposition for obesity or an environment in which calorically dense foods are readily available and physical activity is limited [46]. An interpersonal dimension of obesity is also introduced to this model, by highlighting that general negative public perception of large body size makes larger individuals more likely to be dissatisfied with their own body [47] underpinning isolation and a general lack of social support. Furthermore, Marks also outlines the influential role significant others have on one’s eating behaviour through his consideration of the role of attachment and parenting which highlights a need to understand obesity and its onset from a life span perspective [48–50].

The notion of behaviour being regulated by homeostasis where people should be motivated to eat when hungry and stop when satiated is intuitive, but many people find it challenging to regulate their eating behaviours and to sustain this over a long period of time. A key aspect of tackling obesity is determining the psychological factors that make some individuals more resilient to relapse after initiation of behaviour change (such as autonomy and competency) and how these processes relate to motivation to regulate eating behaviour over time [51, 52]. Even though this has not been specifically addressed in Marks’ COD model, his model highlights this notion of habitual coping which develops over time and the role of life span, suggesting that more consideration of patients and their eating behaviour across the life course may be of use. This raises food for thought as his time frame contrasts with the time-limited approach of bariatric interventions to treat morbid obesity. Furthermore, addressing alternative coping mechanisms beyond the potential of comfort eating requires investigation.

Overall, the outlined theories highlight gaps in the theoretical domain of obesity which as a consequence limit the development of a wider range of health interventions that may work effectively over the long term. In addition, there is a need to understand the function eating behaviour serves for obese individuals in order to redefine or replace this behaviour after bariatric surgery.

## Psychological health in bariatric surgery candidates

It is clear that the development and maintenance of morbid obesity is psychologically complex. However, not everyone living with morbid obesity will opt for bariatric surgery as a method of weight loss, even if they are eligible. This has prompted further research into the psychological co-morbidities of those opting for bariatric surgery. Specific literature on bariatric surgery indicates a higher prevalence of psychological co-morbidities such as mood disorders, eating behaviour disorders and psychological distress in bariatric surgery candidates [53, 54] along with anxiety, personality disorders, alcohol use and low self-esteem when compared to controls or other obese patients who do not seek the procedure [55–57].

Psychological screening before bariatric surgery is commonly used to identify potential contraindications to surgery and additional education or psychological need before surgery, in order to optimise outcomes [58]. However, there has been controversy around active exclusion of bariatric surgery candidates due to psychiatric disorders, with researchers pointing out that these individuals could still experience improvement of health status and well-being postoperatively if adequate support is provided after bariatric surgery [59].

It is important to understand the relationship between obesity and mental health. Despite the greater effectiveness of bariatric surgery compared to other obesity interventions in relation to improved medical outcomes [19], research around persisting psychological issues after surgery is sparse. This may be due to a general lack of postoperative psychological monitoring, in contrast to the amount of screening for psychological disorder and risk before the procedure. Assessing psychological outcomes after surgery in this patient group is important in order to effectively evaluate whether this surgical treatment approach can facilitate resolution of pre-existing psychological conditions that may support recovery. This paper describes three psychosocial outcomes of bariatric surgery, namely psychosocial health, eating behaviours and body image. These outcomes have been selected because of their prevalence in bariatric surgery candidates and the potential influence they have on weight loss success and maintenance.

### Impact of bariatric surgery on psychosocial health

Research has reported improvements in psychosocial status following bariatric surgery including social relations and employment opportunities [60], and improved quality of life [61]. However, although evidence from recent systematic reviews in this area shows that the surgery can result in drastic weight loss and maintenance [19], most of this data is limited to the first 2–3 years of postsurgery follow-up [62••]. Specifically looking at depression, De Zwaan and colleagues [63] investigated the course of anxiety and depressive disorders in 107 extremely obese bariatric surgery patients using face-to-face interviews conducted before surgery and postoperatively at 6–12 months and 24–36 months. Although prevalence of depressive disorders decreased significantly after surgery in their cohort, participants with both depressive and anxiety disorders at baseline lost significantly less weight after surgery. Moreover, postoperative depressive disorder was negatively associated with weight loss at 24–36-month follow-up. Overall, their suggested that presence of depressive disorders after bariatric surgery significantly predicted attenuated postsurgical improvements, inferring a need for clinical attention where postoperative depression is present. More recent research investigating the impact of bariatric surgery on depression has found modest reductions over the initial postoperative years i.e. approximately 2 years [64, 65]. However, subsequent elevations in depressive symptoms in longer term follow-up [64, 66].

Further longer term studies suggest minimal improvements in mental components of quality of life and psychosocial well-being after surgery compared to behavioural interventions and usual care despite overall significant improvements in physical quality of life, weight loss and co-morbidities [62••, 67•]. This finding of persistent mental health problems, regardless of weight loss, compared to counterparts who received behavioural intervention as morbid obesity treatment, suggests a subset within the bariatric surgery patient community that do not do well psychologically despite generally positive medical and physiological outcomes [62••, 24]. Moreover, this evidence emphasises the need for further research in this area to provide more comprehensive understanding of long-term psychological well-being postsurgery.

### Impact of bariatric surgery on eating disorders

Studies have shown that eating problems like Binge Eating Disorder (BED) have a prevalence of 10 to 27% in pre-surgical candidates [68, 69]. Another eating disorder found to be more prevalent amongst this population is Night Eating Syndrome (NES), a core feature of which is a shift in the circadian pattern of eating, resulting in frequent night awakenings linked to nocturnal eating and morning anorexia [70]. As bariatric surgery imposes a physical change in individuals’ ability to consume large quantities and types of food, an important element may be how the procedure affects the complex pattern of eating behaviour. Research implies that the procedure triggers biological changes in the release of gastrointestinal hormones that control appetite which could in turn influence eating behaviour postoperatively [71].

Some studies have found that BED prevalence in pre-surgical candidates persists after bariatric surgery with patients showing either a return to loss of control over eating and binge eating [72, 73], development of frequent eating, labelled ‘grazing’ [74] which as a consequence negatively affect weight loss and weight loss maintenance following bariatric surgery [55, 72]. Interestingly, Wood and Ogden [75] who looked at binge eating behaviour before and after gastric banding in 49 patients found that decreased binge eating as a consequence of having surgery significantly predicted postoperative weight loss. They suggested that the procedure possibly facilitates a change in cognitions relating to food by changing the association between emotions and food. Other studies have similarly described lower hedonic responses to food after surgery, attributing it to lower activation in the brain reward system outlined in the addiction theory [76] and changes in taste perception [77]. Wood and Ogden [78] subsequently identified behavioural intentions as key predictors of reduced binge eating after surgery. This suggests that individuals who present with binge eating at preoperative screening could optimise positive weight loss outcomes if interventions focused on increasing preoperative levels of intention to follow the postoperative eating guidelines.

Studying the impact of NES on bariatric surgical outcomes is similarly important, as postoperative continuation of this eating disorder may hamper weight loss success or maintenance. Despite its high prevalence amongst bariatric surgery candidates, research looking at postsurgical continuation or change in NES-related behaviour is limited [79]. A recent review on NES in bariatric surgery patients implied a decrease in symptoms of NES after weight loss surgery [80]. De Zwaan and colleagues also found no evidence for negative impact on weight loss following surgery due to pre-surgery NES. However, several limitations were noted, such as inconsistency in diagnostic criteria. Moreover, very few studies examined night eating prospectively or followed samples long enough after bariatric surgery to fully examine the impact of NES. Ultimately, more prospective and longitudinal studies looking at the course of this eating disorder, using clear criteria and standardised assessment instruments, are required.

### Impact of bariatric surgery on body image

Body image is a multifaceted construct defined as ‘one’s body-related self-perceptions and self-attitudes, including thoughts, beliefs, feelings and behaviours’ [81]. One construct, body image dissatisfaction, defined as a persons’ negative thoughts and feelings about his or her body [82], is reported as one of the most consistent outcomes of obesity. As previously noted, body image dissatisfaction is positively associated with increased BMI [83, 84] and related to issues such as binge eating and overestimation of body size in obese populations [85]. As such, it is reported as a key motivator for seeking bariatric surgery, especially amongst women [86]. Bariatric surgery has the potential to improve a person’s body image because weight loss is capable of moving them closer to societies prevailing slim ideal. However, the procedure can result in significant changes to appearance such as scarring, sagging skin and soft tissue excess [87] which can in turn, significantly impact upon a individual’s body image.

Despite the significant changes to appearance following bariatric surgery, the impact it has upon patients’ body image has received little attention. Existing research reports positive effects of bariatric surgery upon body shape preoccupation [88–90] body image quality of life [91] attitude towards one’s body [92] and satisfaction with one’s appearance [91]; however, these body image improvements often fail to reach population norms [92, 93]. If negative constructs of body image, such as body dissatisfaction, are positively associated with BMI, it is logical to assume that body image improvements would be related to the amount of weight loss. Indeed, satisfaction with one’s appearance [88] and body image quality of life [94] have been positively correlated with the amount of weight loss. Whilst De Panfilis [95] found that the reduction in body image dissatisfaction observed in their sample of morbidly obese patients did not involve concomitant weight loss, they also found other mediating factors, such as binge eating behaviour, were influential. It appears that body image improvement could be related to patients’ changing attitudes or behaviours following surgery, rather than actual weight loss, but additional investigation to clarify this is required.

Much of the literature reporting improvements in body image investigate short-term changes typically within 2-year postbariatric period [94]. Research that investigates longer term body image after bariatric surgery is important, as rapid weight loss occurs in the first 6 months and then slows down or is even regained [96] after which body image improvements could cease, or concerns could return or change focus. Perhaps one key indicator of long-term postoperative body dissatisfaction is the large number of patients who request body contouring surgery to ameliorate functional and/or aesthetic concerns most commonly related to excess skin. Such concerns can be a long-term burden due to the notable disparity between those who desire it and those that receive it [97]. Body contouring is reported to improve body image following bariatric surgery [87, 97, 98]. However, patients can have high expectation of contouring procedures to improve their appearance [98] and body dissatisfaction may shift to a different part of the body after the procedure. For example, Song and colleagues found that after contouring, body image satisfaction in patients improved regionally, particularly where treatment occurred. This resulted in a shift in body dissatisfaction focused towards previously hidden areas of deformity or other untreated areas that looked visibly disproportionate to the contoured areas [87].

## Conclusions

The literature reviewed suggests that despite drastic weight loss and positive physical health improvements experienced postoperatively over time, some psychological problems, probably linked to a disordered relationship with food [55, 72, 73, 99] present in obese individuals from onset, remain. The findings also highlight the importance of identifying risk groups among bariatric surgery patients who may require additional support with dietary and psychological follow-up [100••].

The superiority of bariatric surgery in improving medical outcomes of the severely obese when compared to other weight reduction interventions remains undisputed [18, 19]. However, at present, our understanding of psychological health outcomes following bariatric surgery is limited. A key reason for this may be the acute biomedical nature in which this surgical intervention for morbid obesity is delivered and assessed. This might be because bariatric surgery and its outcomes are still very much framed within a surgical perspective, making psychological outcomes and time frames less of a priority [101]. This approach seems to contrast with the onset of obesity within the biopsychosocial framework of not only biological attributes but also psychosocial and environmental factors.

A general lack of postoperative psychological follow-up means that very little is known about the effect bariatric surgery has on patients’ psychological outcomes. This is unfortunate considering the array of postsurgical psychosocial challenges the procedure elicits as a result of drastic weight loss and other physiological changes [102], namely body image concerns, mood changes, stress, substance use [94] and weight regain [103]. Research addressing patients’ psychological postoperative needs could reduce the risk of weight regain [104] and optimise the effect of the procedure itself. On a broader level, research that considers the long-term outcomes beyond 2 years is needed to better understand how psychology and surgery interrelate within a behaviour that has developed across the life course. Framing this interrelation is crucial, as weight loss and other potentially relevant behavioural changes occur gradually and therefore warrant long-term monitoring.

Finally, existing research into bariatric surgery is generally quantitative, with most outcomes focused on physiological measures such as weight and obesity co-morbid medical issues. Existing psychological research relies heavily on self-report quantitative data, which does not allow the opportunity to adequately capture detailed insights into the experience of having bariatric surgery from a patient perspective, suggesting further requirement for qualitative research. Rigid responses acquired through self-report measures make it difficult to collect data over a long period of time that adequately encapsulates the disease trajectory of morbid obesity alongside psychological experience. This limitation may have led to a lack of theory building around morbid obesity found in this review.
